# Depth perception in disparity-defined objects: finding the balance between averaging and segregation

**DOI:** 10.1098/rstb.2015.0258

**Published:** 2016-06-19

**Authors:** P. Cammack, J. M. Harris

**Affiliations:** School of Psychology and Neuroscience, University of St Andrews, St Andrews KY16 9JP, UK

**Keywords:** stereopsis, binocular disparity, depth perception, disparity averaging, object segregation, psychophysics

## Abstract

Deciding what constitutes an object, and what background, is an essential task for the visual system. This presents a conundrum: averaging over the visual scene is required to obtain a precise signal for object segregation, but segregation is required to define the region over which averaging should take place. Depth, obtained via binocular disparity (the differences between two eyes’ views), could help with segregation by enabling identification of object and background via differences in depth. Here, we explore depth perception in disparity-defined objects. We show that a simple object segregation rule, followed by averaging over that segregated area, can account for depth estimation errors. To do this, we compared objects with smoothly varying depth edges to those with sharp depth edges, and found that perceived peak depth was reduced for the former. A computational model used a rule based on object shape to segregate and average over a central portion of the object, and was able to emulate the reduction in perceived depth. We also demonstrated that the segregated area is not predefined but is dependent on the object shape. We discuss how this segregation strategy could be employed by animals seeking to deter binocular predators.

This article is part of the themed issue ‘Vision in our three-dimensional world’.

## 1. Introduction

Binocular disparity, the tiny differences between right and left eye views of a scene, can be used to segregate an object from its background even without other visual information about the boundary between object and background. This was first popularized by Julesz in 1971 via the random dot stereogram (RDS) [1], a stimulus that contains disparity information without other form cues. Julesz used RDSs to suggest that binocular vision alone can break camouflage, as disparity reveals the three-dimensional shape of an object even when the object has identical patterning to the background. Thus, disparity can break camouflage designed to make an object match its background in luminance or colour, a common evolutionary strategy [2–4]. Evidence to support the specific suggestion is scant, although several studies have shown that masking, where an object is harder to see when it is superimposed on another scene, is reduced when target and mask have different disparities [5–7].

One can think of the process of obtaining depth from disparity as having at least two stages. The first is disparity extraction, of which we now know a great deal, including upper and lower disparity limits that can be linked to the properties of disparity sensitive neurons [8–12]. Disparity extraction is thought to rely on a process akin to local cross-correlation, where individual disparity-sensitive neurons signal a single disparity over a spatial region—their receptive field [11,13–18]. Models of this process can explain a variety of effects, including why some transparent scenes are perceived as a single plane rather than a pair of (or more) planes at different depths [19–22]. However, these models are designed to explain how disparity is extracted; they do not consider the potentially different problem of how the extracted disparities (which may not be extracted veridically) are combined across scale, space and time to represent depth.

The disparity combination process is much less well explored, but we know the visual system is prone to error here. For example, disparity averaging is thought to be partially responsible for our perception of interpolated depth, across regions where no disparity exists [23–25]. Additionally, there exist depth–contrast effects, where the depth of nearby objects or stimulus regions can affect perceived depth of a target area [26,27].

When combining disparities across space for depth perception, there are two potentially opposing aims for the visual system. Extraction of disparity will not always be veridical: by averaging extracted disparities across space, it is possible to improve the signal-to-noise ratio and more accurately estimate overall depth at some location. However, too much averaging will effectively smooth over potentially important depth edges, resulting in inaccurate depth appearance and reduced effectiveness of depth segregation. One key question is how the visual system balances the need to average with the need to precisely represent fine-scale depth information. Our aim here is to explore this problem.

**Figure 1.**
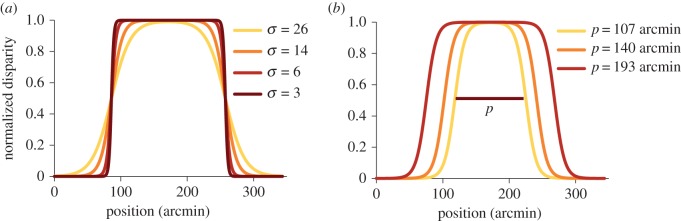
(*a*) Cross section shows depth as a function of width of the stimulus used, for several different smoothness coefficients (*σ*). Peak disparities were between 5.4 and 8.4 arcmin, the *y*-axis shows normalized disparity (disparity at each location/peak disparity for that trial). (*b*) Cross section of the stimulus used in experiment 3, showing the effect of manipulating plateau size (*p*).

**Figure 2.**
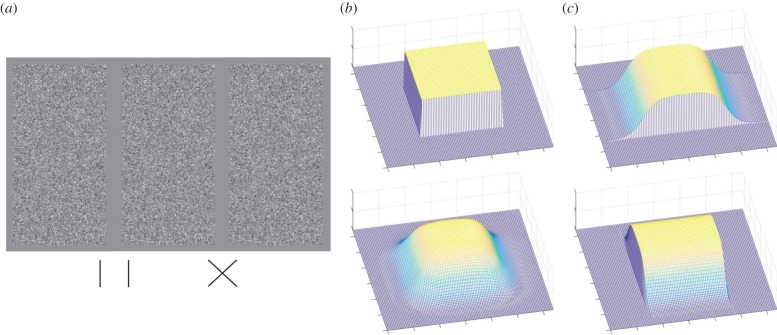
(*a*) Stimulus from experiment 1 set-up for divergent (left) and convergent (right) free fusion. (*b,c*) Three-dimensional representation of stimuli in experiment 1 and 2. In this cartoon representation, the dark blue background mesh is in the plane of the screen, lighter colours demonstrate depth extending out from the screen towards the observer.

In this study, we used RDSs to measure perception of depth in objects containing either a sharp or smooth gradation in disparity, from the background to the peak depth at the object centre (figures 1 and 2). As our aim was to study effects caused by disparity combination, and not disparity extraction, we used a large spatially depth-defined region (2.8°), and easily visible peak disparity (5.7 arcmin) whose dominant depth corrugation frequency (around 0.17 cpd) was in the range of high sensitivity and large upper disparity limit [12,28]. We assumed that disparity would be extracted veridically for this range.^1^

We also assumed that the visual system must identify a boundary, i.e. segregate a region, before averaging to improve that signal. We reasoned that, for an object with a sharp disparity edge, there would be a strong signal defining that edge across all spatial scales of disparity extraction. This could serve to form a boundary for any subsequent disparity averaging across the object (to improve signal-to-noise). For an object with a smooth depth edge, the disparity along the edge changes continuously, making the boundary less well defined. However, the visual system will still need to have a ‘rule’ for segregation somewhere along this continuous edge. Note that here we are not proposing an alternative model to that accepted for disparity extraction [12–17]. Rather, we are taking a different approach to explore how extracted disparities are combined. Our aim was to test how far we could go to explain human depth perception, assuming veridical disparity extraction, with errors caused by failures in disparity averaging and segregation.

We explored the segregation rule by measuring depth sensitivity and perceived depth. In experiment 1, we measured the bias and sensitivity in assessing the peak depth of a smooth object with a constant width at half-depth, compared with an object defined by an abrupt change in depth (figure 2*b,* bottom and top, respectively). Ideally, averaging should be applied to regions likely to be of the same depth. This is well defined for a sharp-edged object. For the smooth-edged object, averaging could take place over the central region of constant disparity, or from a region starting at some point between the peak and the background. If the latter, we expect a decrease in perceived peak depth. We also considered the potential influence of half-occlusions in experiment 2. These are areas of a scene where the depth edge is so sharp that the foreground occludes one eye's view of the background (figure 4*a* and see also [29]). Based on the experimental results, we proposed a model to describe the segregation rule used, and the model was tested further in experiment 3.

## 2. Methods

### (a) Apparatus

Left- and right-eye images were presented side by side on a luminance calibrated CRT monitor (Iiyama HM204DT A Vision Master Pro 514) in a darkened room. Stereoscopic presentation was achieved using a Wheatstone stereoscope. Experiments were coded using MATLAB^®^ (2013) with stimuli displayed using the Psychophysics Toolbox [30,31]. A chin rest was used to stabilize viewing position (1 m from the screen), and each participant adjusted the central stereoscope mirrors to obtain comfortable fusion. Responses were made using the up and down arrow buttons on a keyboard, and the spacebar was used to initiate stimulus display.

### (b) Stimuli

Random dot stereograms [1] were used to isolate the binocular disparity cue, so that there was no other information about object edges and depth. The screen (23° × 17°) was mid-grey (6.1 cd m^−2^). An RDS (5.6° wide by 11.2° tall) was filled with black (less than 0.01 cd m^−2^) and white (12.24 cd m^−2^) dots of size 2.14 × 2.14 arcmin, randomly distributed at a density of 326 dots per square degree (a Nyquist frequency of 9 cpd [11]). Within each RDS, there was a pair of depth-defined patches, one above the other, each containing depth projecting towards the observer from the plane of the screen (figure 2*a*). For all experiments, the standard patch was a square of side 2.8°. Standard patch location was randomly assigned to either the upper or lower location on the screen. Standard patches contained a sharp transition from background depth to the foreground, so that all pixels in the depth-defined region had either zero or the peak disparity. We call this the sharp-edged object (figure 2*a,b*, top). The crossed disparity of the sharp-edged object was constant at 5.7 arcmin (participants were not informed that the standard patch was of constant disparity). In the right eye's view, when the foreground was shifted to the left to deliver disparity, there was a region on the left of the object where the dots of the foreground overlapped the background, whereas a small rectangular gap remained on the right. To avoid this providing a monocular cue to shape, the overlapping background dots were deleted and randomly reassigned positions in the empty rectangle. This process was repeated in the left eye. This created regions of the background that only one eye could see, called half occlusions (HOs).

The test patch was given a different disparity profile (figure 1) to produce a smooth change in depth. It contained at least two depth edges that had a smooth transition between the background disparity and the peak disparity of the object, although the exact shape of the object was different for each experiment. The shape of the smooth edge was defined by2.1
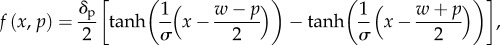
where *f*(*x*) is the *x*-axis disparity contribution at any point (*x*, *y*), *δ*_p_ is the peak disparity of the stimulus, *w* is the width of the object, *p* is the full width at half maximum depth (referred to as plateau size) of the object and *σ* is the smoothness coefficient. The variation in shape with *σ* for experiments 1 and 2 is shown in figure 1*a*. The range of crossed peak disparities in the smooth test object could vary from 5.4 to 8.4 arcmin for experiments 1 and 2 and 4–10 arcmin for experiment 3 (details below). On each trial, the test object was given a peak disparity either drawn from these extremes or one of five intermediate disparities. Figure 1*a* shows normalized disparity (disparity at any location divided by peak disparity for a particular trial) as a function of position, to illustrate how a higher value of smoothness coefficient indicates a smoother edge with a lower disparity gradient and rate of change of gradient.

We ensured that the smoothness coefficient could not be so high that the disparity at the peak of the object was less than 0.99 *δ*_p_. Additionally, the maximum gradient was not allowed to be large enough to deliver HOs [29] or be above the disparity-gradient limit [32]. The function in equation 2.1 was chosen as it is easy to manipulate, and the average depth of the whole object was half the peak depth (for the range of smoothness coefficients used).

The shape of object defined by equation 2.1 has two key variables. The smoothness coefficient, *σ*, varies the shape of the object as shown in figure 1*a*. Changing the smoothness coefficient does not change the average disparity across the whole object or the position of the disparity inflection points. The second key variable is the plateau size *p*. This took a constant value of 171 arcmin in experiments 1 and 2, but varied in experiment 3. Varying this moves the inflection points of the function closer together/further apart (figure 1*b*), changing the width or height of the smooth object. Plateau size is a particularly interesting variable as it coincides with three major properties of the smooth object which could be used in segregation of an edge for the object. First, it corresponds to the distance between the inflection points on the function. Second, its width defines the points of maximum gradient of the object, and third, it is the separation between locations that have half the peak depth (i.e. *δ*_p_/2), which is also the average depth of the object. Plateau size was varied in the third experiment, where we were interested in testing if averaging is based on the size of the object. Because the average disparity of the object changes systematically with changing plateau size, we can predict how the perceived depth of the peak is affected by changing object shape and compare it with psychophysical measurements.

*Experiment 1:* test objects contained a smooth depth discontinuity (figure 2*a,b*, bottom). We call this the smooth-edged object. The plateau width was half the width of the object, equal to 171 arcmin (*p* = *w*/2), and the average disparity of the object was constant at half the peak depth of the object. The shape was defined by2.2
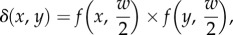


where *δ(x, y)* is the disparity at the point (*x*, *y*). Test stimuli contained one of four smoothness coefficients (3, 6, 14 and 23: unit is per pixels, where 1.073 arcmin = 1 pixel).

*Experiment 2:* test objects contained a combination of smooth and sharp edges, with three smoothness coefficients (3, 14 and 23). In the first condition, the left/right edges of the object were smooth, and the top/bottom edges sharp (figure 2*c*, top). Sharp edges along horizontal borders do not deliver HOs, so no half-occlusions (NHOs) were present in this condition. In the second condition, the shape was rotated through 90°, so that the left/right edges were sharp and the top/bottom smooth, resulting in HOs at the left/right edges (figure 2*c*, bottom). The disparity *δ* of a dot located at (*x*, *y*) in this stimulus was described by2.3
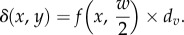


The first term is the equation for the smooth edge (equation (2.1), here orientated along the *x*-axis, thus causing NHOs). *d_v_* is the disparity contribution, where
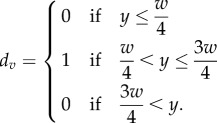


When the *y* coordinate of each stimulus dot lay within the central region of the stimulus, the disparity contribution *d_v_* was 1 and the magnitude of a dot's disparity was dominated by the equation for the continuous edge. When the *y*-coordinate was outside the sharp edge, the entire equation reduced to 0 for all *x*, so there was zero disparity within this region. This object had fewer dots with disparities that were neither zero or *δ*_p_ than in experiment 1 owing to the removal of the second continuous edge.

*Experiment 3:* the smooth test patch from the first experiment was altered to allow a change in the plateau or half-depth (where *δ* = 0.5 *δ*_p_) independently of the edge shape and object size (figure 1*b*). We were primarily interested in the effect on perceived peak depth of the plateau size *p* (the separation between the edges of the patch at half-depth).2.4
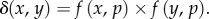


Only one smoothness coefficient was used (14). The plateau size was set to three different values: 107, 140 and 193 arcmin.

### (c) Participants

Participants were recruited via the University of St Andrews' online recruitment service and were recompensed for their time. Stereoacuity was tested with a TNO test [33]. Those who could not correctly identify a baseline depth of 8 arcmin or larger were excluded from the study. This is a conservatively high choice for the disparity threshold for exclusion. Naive observer thresholds vary widely in a dimly lit room, and we wanted to exclude as few participants as possible. The majority of participants were included/excluded based on the performance on a further demonstration, which directly tested their ability on the task [34].

### (d) Procedure and analysis

The task and stimulus shape was initially explained using a cross-sectional line drawing (*x–z* plane) of an artificial stimulus. Participants were informed that the maximum depth or peak was always in the centre of the object and that this was what they would be asked to report on. A screenshot of the experimental stimuli was then presented to the participants through the stereoscope. To ensure participants could correctly see the stimulus, they were asked to describe the shapes present in the stimulus. If the participant used ‘height’ instead of ‘depth’ when self-describing the object, then this was accepted.

Each participant then completed a shortened demonstration version of the experiment, using a two-alternative forced choice design, with the standard and test stimuli presented above one another, to familiarize them with the task. In the demonstration, larger disparities were used (maximum 9 arcmin crossed), and the stimulus was initially shown for 10 s, reducing to 2 s by the 10th trial. Participants were asked to indicate if either the ‘upper or lower stimulus had a greater peak depth’ and specifically instructed to ignore the surrounding shape of the object. After completing the demonstration run, we checked the data to ensure the participants understood the task before they were allowed to continue the experiment. If they did not, then we tried to ascertain what they had misunderstood and correct this, then re-ran and re-checked the data. If the misunderstanding could not be specifically identified, or their second run did not show an improvement, then the participant was excluded from further study (four participants were excluded in this manner).

Every experiment followed the same procedure: a fixation cross at the centre of the screen (black less than 0.01 cd m^−2^ on mid grey 6.1 cd m^−2^, 69 arcmin wide/high) appeared until the space bar was pressed. The stimulus was then presented for 2 s, followed by a response prompt screen: black text on mid grey requested participants to press either the up or down arrows to indicate which stimulus had a greater peak depth. The prompt screen was displayed until a response was given. The fixation cross was then redisplayed, and the next trial initiated by button press. Trials were presented in blocks (approx. 300 trials) that took around 10–15 min to complete, with a clear break between blocks. No participant spent more than 1 h participating on any 1 day.

We used a method of constant stimuli design to explore how the shape or size of the depth edge affected peak perceived depth. We collected data from a minimum of 70 trials (maximum 91) for each peak depth. This allowed us to plot a full psychometric function: the proportion of standard objects chosen as deeper, as a function of the displayed peak disparity [35]. A cumulative normal was fitted [36], and the point of subjective equality (PSE) extracted from the fitted function. Thresholds, a measure of the slope of the fitted function, were defined as half the difference between the disparity values at the 75% and 25% points on the fitted function.

## 3. Results

### (a) Experiment 1: perceived peak depth as edge profile changes

**Figure 3.**
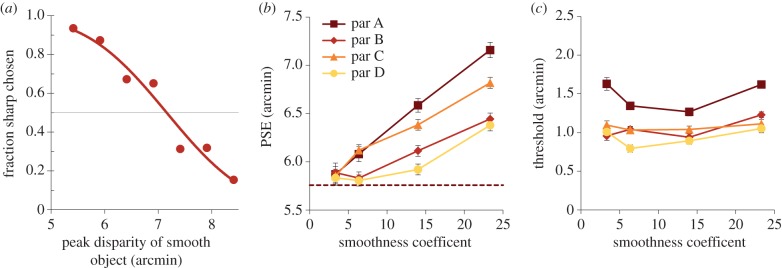
(*a*) Raw data and example psychometric function for one participant. (*b*) PSEs for all four participants as a function of smoothness coefficient. The dotted horizontal line shows the peak depth of the standard, sharp-edged, object. (*c*) Thresholds for four participants. Error bars show standard error of the mean.

Here we sought to test if perceived depth varied as the depth profile of the disparity-defined object edge was varied. Figure 3*a* shows raw data for one of five participants, and an example fitted psychometric function, where the participant's responses are plotted as a function of the peak disparity of the smooth object (full psychometric functions for all observers are in the electronic supplementary material). For one participant, the psychometric function was very flat, and the extracted PSE was outside of the displayed range of disparities (5.4–8.4 arcmin) suggesting this observer to be very poor at the task. This participant's data were omitted from further study.

Figure 3*b* shows PSEs for the four remaining participants as a function of smoothness coefficient (larger coefficients represent a smoother depth profile). A repeated-measures ANOVA showed a significant effect of smoothness coefficient on the observed PSEs (*F*_3,9_ = 21.1, *p* < 0.0005). A greater smoothness coefficient delivered a larger PSE, thus smoother-edged objects were perceived as having a smaller peak depth than the sharp-edged object, for the same physical depth.

Figure 3*c* shows the extracted thresholds as a function of smoothness. A Bonferroni pairwise comparison showed no significant effects between any smoothness values on the observed thresholds (*p* > 0.1).

These results are rather surprising, there are many elements in the central area of the stimulus that are located at the peak disparity. The fact that the visual system cannot correctly compare the peak depths of the two objects indicates that it is unable to obtain the peak depth of the object independently from its overall shape. The effect is notable because it takes place over such large length scales (over 80 arcmin).

There are two reasons why perceived peak depth might be smaller for the smooth-edged object: (i) disparity is averaged across the whole (or part of the) object to improve signal/noise ratio; (ii) the HOs present in the sharp-edged object might provide an additional cue to depth and deliver greater perceived depth. We tested the latter idea in experiment 2.

### (b) Experiment 2: enhanced depth from half-occlusions

**Figure 4.**
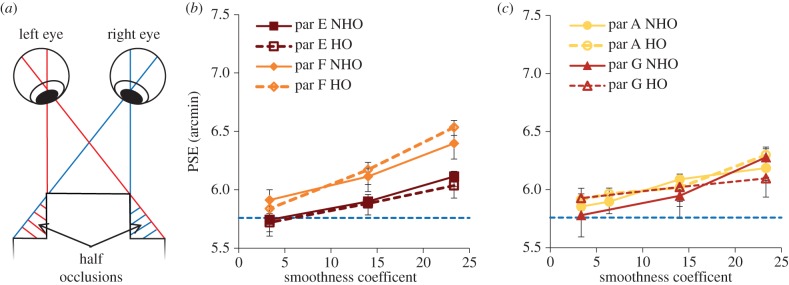
(*a*) Diagram shows half occlusions (HOs; hatched regions) when an observer views a patch standing out in depth from a background (solid black lines). (*b,c*) PSEs for all four participants as a function of smoothness coefficient. Solid lines are for no half occlusions (NHO) and dashed for HOs conditions. The dotted blue horizontal line shows the peak depth of the sharp-edged object. Error bars are 1 standard error.

Here we tested whether HOs such as those shown in figure 4*a* were responsible for there being more depth perceived in the sharp-edged object by comparing a condition with sharp vertical edges and smooth horizontal edges (creating HOs) with a condition where the patch was sharp-edged for horizontal edges (NHOs).

Two of six naive participants were unable to complete the fifth plate of the TNO test (more than 8 arcmin threshold) and had PSEs that were outside of the measured range of disparities, so were excluded from further study (data available in §3 of the electronic supplementary material). Participant A had previously participated in experiment 1, but changes in performance between experiment 1 and 2 are unlikely to be practice effects as their performance did not improve as they completed additional blocks. Figure 4*b*,*c* shows PSE as a function of smoothness coefficient for the four participants for both conditions. The data confirm that, as for experiment 1, observed PSE was higher for the larger smoothness coefficients. A repeated-measures ANOVA showed there was no significant difference between PSEs from the HO and NHO conditions (*F*_1,3_ = 0.452, *p* = 0.459) or the thresholds (*F*_1,3_ = 0.001, *p* = 0.975). Thus, we have no evidence that HOs contribute to the bias in perceived depth found in experiment 1.

Note that the bias in perceived peak depth appeared a little lower here than that found in experiment 1 (compare figures 3*b* with 4*b* and 4*c*). We did use different observers here, so this effect could be due to individual differences. However, the object presented here had only one pair of smooth edges, so the smaller bias might suggest that the range of presented disparities is influencing perceived peak depth. In §3c, we implemented a model inspired by this possibility.

Thresholds for this experiment showed variation between participants, but for all smoothness coefficients and both conditions, the thresholds did not vary significantly (repeated-measures ANOVA, *F*_1,3_ = 0.001, *p* = 0.975). Mean threshold for all participants for the HO condition was 1.06 arcmin and for the NHO condition 1.08 arcmin. There were large differences between participants, but each participant showed consistent thresholds for all conditions to within 0.2 arcmin. These results suggest that the reduction in perceived peak depth for the smoother objects is not related to the presence of HOs.

### (c) Modelling

The results of experiment 1 were rather surprising in that there is a large region (a square area with side length over 80 arcmin where elements have disparities over 95% of the peak disparity) at the centre of each stimulus specifying the peak depth. The visual system is clearly unable to use that information alone. Estimates of peak depth were smaller than veridical, suggesting that averaging, or some other combination, must be going on at a rather large scale.

Averaging of disparities will necessarily take place at the disparity extraction stage: current models of disparity extraction essentially rely on cross-correlation, which requires averaging across small regions of a scene [15–18]. If averaging is the cause of the fall in perceived peak depth with smoothness in our data, then it must occur over a large region as there is a large central area where elements are located at the peak disparity. We reasoned that this would be a much larger region than current models of disparity extraction could account for. To test our reasoning, we implemented a simple disparity cross-correlation model, operating over a number of spatial scales.

The cross-correlational model took screenshots from the stimulus presented to the observer, and cross-correlated small square regions, or windows (from 10.7 to 85.6 arcmin) from the left eye's image with the right eye's image. For each location in the left eye's image, cross-correlations were performed for a range of horizontal offsets of the window in the right eye's image. The disparity for this location was defined as the horizontal offset with the maximum response of the cross-correlator. This process was repeated across all horizontal and vertical locations, for each window size. For each window size, we calculated the disparity at each point in the image.

We ran this simple model for disparity-defined objects with smoothness coefficients 0, 14 and 26, all with a peak disparity of 6.0 arcmin. For all window sizes, our simple cross-correlation model veridically extracted a disparity of 6.0 arcmin as the peak depth across a large central region of the object; the exact size and shape of this region varied with window size, but was typically around 60 arcmin across. Thus, cross-correlation across a range of scales (window sizes) did not deliver peak disparities that were different from those assigned in the stimuli. This was not surprising as the depth corrugations in our stimuli were very coarse, equivalent to around 0.17 cycles per degree of disparity corrugation, well below disparity frequencies where stereoresolution breaks down [11,16,28].

As our simple cross-correlational model did not account for our results, we considered the effect of further averaging occurring at a later stage, where extracted disparities are combined to form a depth representation. We developed a simple descriptive model to explore if, and how, the visual system averages extracted disparities across an object to obtain a depth estimate. The model assumed that the visual system first uses the extracted disparities to segregate the object from its background. The segregated object then defines the shape and size of the area across which disparity is averaged to estimate the depth of the object. Averaging only over the segregated area avoids including background disparities that would interfere with foreground depth perception and vice versa, and gives a more reliable depth estimate.

The stimulus consisted of a square object centred in the image. We chose a square region over which to average disparity, centred on the middle of the disparity-defined object. We call this the averaging window. However, we did not know what rules the visual system might use to segregate between the object and background, so we let the data tell us, by exploring what size of window would best fit our data.

Each stimulus patch in experiment 1 contained a region where elements had non-zero disparity, as defined by equation (2.1). The modelling was based directly on veridical disparity estimates. This is not to say that we think the visual system veridically estimates disparities of all points in a visual scene, but rather we wanted to see how well a simple model of disparity combination could explain our results.

In order to calculate the summed disparity within the square region, *δ*_region_, we applied disparity averaging over a square window of size (*x*_2_ − *x*_1_, *y*_2_ − *y*_1_), where *x*_2_ = *y*_2_ and *x*_1_ = *y*_1_, by integrating the disparity function (equations (2.1) and (2.3), see electronic supplementary material, §S1 for mathematical details):3.1
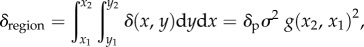


where3.2
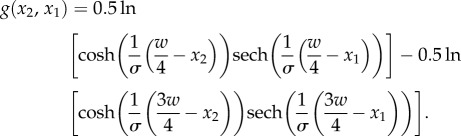


We assumed that peak depth was based on the average disparity over this window, and obtained this by dividing the summed disparity within the whole stimulus patch, *δ*_region_, by the area enclosed by the window3.3
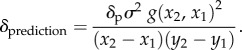
To fit the model to our data, we varied only one parameter—the window size (*x*_2_ − *x*_1_). Essentially, we allow the model to ‘choose’ how to segregate the object from the background. We iterated through different values of window size, calculating the predicted perceived peak depth for all four stimulus smoothness coefficients at each window size. The window size that minimized the reduced chi-squared test statistic (across all smoothnesses) between model output and human data was chosen as the best fit.

**Figure 5.**
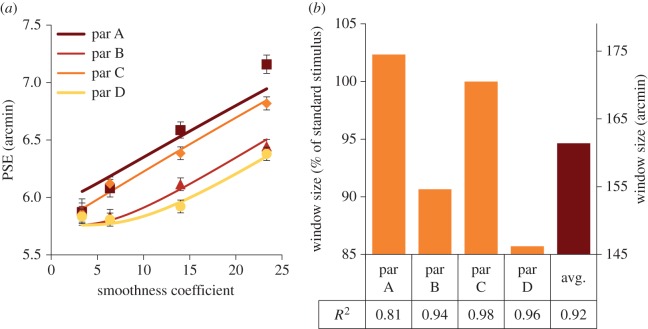
(*a*) PSEs for all four participants are shown by filled symbols, as a function of smoothness coefficient. Solid lines are the model fit. Error bars are 1 standard error. (*b*) Bar chart shows size of fitted window relative to percentage of standard stimulus size and window size; the table below shows *R*-squared value for each fit.

Figure 5*a* shows the experimental data and the best-fit model output (lines through the data in figure 5*a*) for our four observers from experiment 1, with the best-fit window size individually calculated for each observer. The best-fit window sizes for each observer are shown in 5*b* (similar fits for experiment 2 are in §2 of the electronic supplementary material). Overall, the model accounted for 92% of the variance of the data, with the lowest *R*-squared (a *R*-squared of 1 indicates a perfect fit, a *R*-squared of −1 or less indicates that a linear function better fits the data) value being 0.81 and the highest 0.98. A similar process was applied to the data from experiment 2 and a similar result was found (see §2 of the electronic supplementary material).

In principle, the peak-depth task could be performed by obtaining an estimate of the depth at the very centre of each stimulus. Such a simple estimate predicts no difference between stimulus conditions and clearly does not fit our data. Our model assumed that to obtain the best estimate of peak depth, a square region was chosen over which disparities were averaged. The model was well able to fit the data, and the best-fit window was estimated to be of side 162 arcmin. We explored other averaging window shapes to test how the size of the window was related to object shape. Neither a circle of variable radius (see electronic supplementary material, with an *R*-squared = −239 to −15), nor a weighted average with a Gaussian (did not converge), came close to predicting either the size or shape of the flattening. This suggests that the shape of the object is relevant to the shape of the averaging window.

The average window size fitted by the model was 162 arcmin. This is similar to the stimulus plateau size (width/height at half-depth) for all the smooth-edged and sharp-edged objects (squares of length 171.2 arcmin). This suggests two possibilities, which will be explored in experiment 3: first, that the visual system chooses the region within which to average based on the shape of the object, with the edges of that region based on the disparity plateau size. We will refer to this model to as ‘half-depth averaging’. The second possibility is that the visual system uses the standard stimulus, which has constant size and shape, as a template to segregate the test stimulus into object and background. We will refer to this as ‘template matching’.

### (d) Experiment 3

Here we tested whether the visual system averaged disparity information based on the shape of the smooth test stimulus (specifically averaging across the plateau of the stimulus), or by using the fixed size standard object as a template.

**Figure 6.**
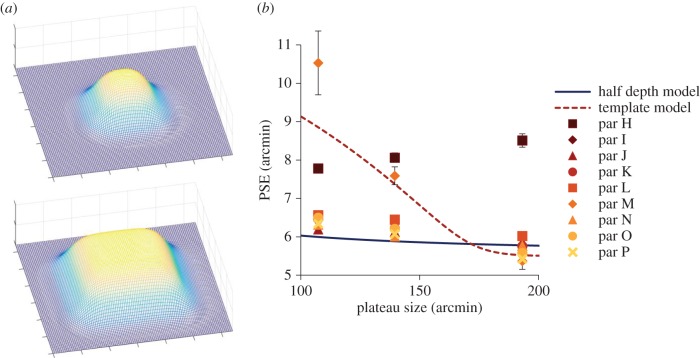
(*a*) Three-dimensional representation of the manipulation of plateau size. (*b*) PSE as a function of plateau size; solid points are PSEs for all participants. The solid line shows the prediction of the half-depth model and the dashed line the prediction of the template model. Error bars are 1 standard error.

We altered the plateau size of the smooth object (the distance between the inflection points): see figure 6*a* for a graphical representation of this manipulation, and figure 1*b* for a cross-sectional view. We ran two versions of the model, which has no free parameters, to obtain predictions for participant performance (expected PSE) if they followed either the template matching or half-depth averaging predictions (mathematical details in electronic supplementary material). These predictions are shown as the dashed red (template) and solid blue (half-depth) lines in figure 6*b*. As the model has no free parameters, there is no flexibility in the model to account for variations in participant's performance, so we expect the model to be unable to fully account for all sources of error. The stimulus was displayed using a larger range of disparities (between 4 and 10 arcmin peak disparity) to ensure that either prediction at 107 arcmin plateau size could be tested.

All 10 naive participants passed the TNO test, although one was excluded owing to delivering a flat psychometric function. Participants compared the smooth-edged stimuli with the standard stimulus, as in experiments 1 and 2, and were asked to judge which had the larger peak depth.

Figure 6*b* shows results for the remaining nine participants. Note that most measured PSEs conform closely to the half-depth model (blue solid line), and are very far from the template prediction (red dotted line). A chi-squared goodness-of-fit test (where a chi-squared of 1 is an optimal fit) indicated that the half-depth model gave an acceptable fit but did not account for all sources of error in all participants, with a chi-squared between 1 and 5.5 for seven participants (excluding participants H at 49 and M at 18). This is considerably better than the performance of the template model, which performed very poorly with a chi-squared between 140 and 276 (excluding participants H and M at 8 and 33, respectively). We should emphasize that the model was fitted with no free parameters, with window size being *fixed* as the distance between the half-depths of the smooth edged object in the half-depth model, or the edge length of the sharp-edged object in the template model. Why specifically the size of the plateau appears to be the governing factor is not clear.

Participant M showed a different pattern of performance. For them, PSEs fell dramatically as plateau size was increased, so their data fell closer to the prediction of the template model, although the test for goodness of fit indicated that this was a poor fit. Participant H had a very unusual response where their PSEs increased with plateau size. Both these participants had thresholds more than three times those of the other participants (par H: 2.3–2.9 arcmin, par M: 7–4 arcmin, all other participants 0.5–1.3 arcmin), indicating that they found this task much harder than other participants.

## 4. Discussion

This paper has addressed a key question in disparity processing: how does the need to average to enhance signal-to-noise ratio interact with the need for edge extraction to enable object segregation? Our aim was to explore how disparity averaging and subsequent depth extraction was affected by the three-dimensional shape of a depth edge defining the object. In experiment 1, we measured the bias in assessing the peak depth of a smooth object compared with an object defined by an abrupt change in binocular disparity. We found that smooth-edged objects were perceived as having a smaller peak depth than sharp-edged objects. However, there is a major difference between the object types, namely the presence or absence of HOs. In experiment 2, we demonstrated that depth biases owing to HOs could not account for the misperception found in experiment 1. We next proposed a model to explore the disparity segregation and combination rule used. The model used the shape of the object to determine the region over which disparities should be averaged, and we found that it described the smaller peak perceived depths found for the smooth-edged objects, and predicted the size of the region over which averaging occurred. A third experiment compared this shape-based averaging model with a very simple template alternative, where the size and shape of the averaging window was dictated by the sharp-edged comparison object. We found that the properties of the smooth-edged object, not the comparison object, dictated the area that was averaged over. The implications of each finding will be described below, in relation to the current literature.

### (a) A role for monocular half-occlusions?

In the first experiment, there was the possibility that the presence of HOs in the standard stimulus could have caused the brain to assume the smooth-edged object was flatter than physically presented. However, in experiment 2, the presentation of a stimulus that could be rotated to be presented with or without a HO showed no significant difference between the half occluding and the non-half occluding condition. Although other studies have found that HOs can contribute to perceived depth ([37,38] or see [29] for an in-detail discussion), we found no evidence that the visual system is using HOs to help assist the peak depth judgement of objects.

### (b) Averaging and segregation versus disparity extraction models

It is well established that disparity estimation is the first major step in the processing of depth from binocular disparity. As described in the Introduction, we now know a lot about this process, and elegant models of it have very powerfully explained a number of perceptual effects [15–18,39]. However, very little work has addressed how the extracted disparity estimates are combined across scale and space to obtain depth perception.

How disparities are combined is a tricky problem to work on, because one can imagine any number of ways that the outputs of disparity correlators could be combined, and there is very little data out there to constrain the problem. The issue is also difficult to address because it is hard to separate the effect of disparity extraction and subsequent combination stages. Here, we worked to study that combination stage alone, by using stimuli where disparities should be veridically extracted. This was backed up with a basic disparity-correlation model that delivered veridical disparities over the parameter ranges we tested. We chose a simple model for how disparity information must be combined: that there must be a choice made about which areas to average over based on the disparity between the foreground and background, and we studied the simplest way this could be achieved.

Thus, our model is not an alternative to the standard models based on combinations of disparity detectors. Rather, we used our simple model to provide a description of the ways in which perception of a three-dimensional scene may be created from the extracted disparity information. We anticipate that future work will use the information from our model as a guide to the way in which disparity detector outputs may be combined when segregating and averaging depth in objects. In §4c, we review experimental literature providing evidence for disparity averaging.

### (c) Perceived depth and disparity averaging

In the literature, there have been a number of different phenomena observed where the perceived depth from disparity does not coincide with reality. Some of these are likely caused by constraints of the disparity extraction stage, but others may not be. For example, perceived depth from binocular disparity is commonly found to be non-veridical in the absence of additional scaling cues to indicate viewing distance [40–42]. As our stimuli were all presented at a single viewing distance, and observers asked to make a relative peak depth judgement between smooth- and sharp-edged objects, mis-scaling of distance cannot account for the apparent compression of perceived depth that was observed for the smooth-edged objects.

There is very little research in the literature that compares the perceived depth of different disparity-defined objects. We know that mandatory disparity averaging occurs across some types of stimuli. This kind of disparity averaging most likely takes place at the disparity extraction stage, where position information is necessarily pooled across space [15–18]. For example, disparity corrugations of more than five cycles per degree are not detected and are thought to be averaged [43]. This is thought to occur, because the finest scale disparity detectors are around 5 arcmin across [10,15]. Any variation in disparity of a finer scale will therefore be averaged across the size of the smallest processing units. However, this is a very much smaller scale than the averaging we are reporting, which appears to be taking place over distances of 100+ arcmin.

Disparity averaging is also reported when two disparity-defined planes overlap (stereo transparency). Kaufman *et al.* [44] were the first to report that depth in a RDS containing a pair of planes is perceived as the average disparity of the two planes, whereas Parker & Yang [20] explored the conditions required to cause averaging. Typically, the percept of two planes breaks down into a perception of a volume defined by dots when the separation between the planes is below 2–6 arcmin [20,21,45]. Although averaging in these studies occurred over a similar range of disparities to those used here, there is a major difference in the lateral separation between dots of different disparity: with overlapping planes adjacent dots were frequently of very different disparities, whereas the dots presented in our stimuli were on a smooth opaque surface where adjacent dots were of similar disparities. Akerstrom & Todd [19] found the difference between opaque and transparent surfaces to be significant, with superior disparity discrimination between two adjacent opaque surfaces than in two overlapping transparent surfaces. Some of the above effects might well be caused by disparity extraction, especially when disparity-defined elements are in close proximity, rather than by subsequent averaging. For example, Harris [46] found that introducing dots at disparities between the planes reduced the perceived depth between the planes further. Modelling of scale-specific disparity extraction showed that some of the effects found could be explained by disparity extraction.

Other research shows that errors in perceived depth are reminiscent of the simultaneous contrast illusions in the brightness domain, and it is harder to attribute them to constraints on disparity extraction. For example, in the Craik–O'Brien–Cornsweet illusion, a pair of equal luminance patches are connected by a region containing an increasing luminance gradient with a step decrease in luminance at the centre. Although of equal luminance, the side patches appear to be different [47–49]. An analogous effect is found with depth edges [26], and the effect is larger for shallower disparity gradients [50]. The effect has been explained in terms of the visual system being relatively insensitive to the shallow depth gradients [26,50], but could be thought of in terms of disparity averaging across specific stimulus regions.

Our results show that there appears to be long-range depth averaging across objects where the borders of the averaging are defined by the properties of the object itself. Our results are akin to findings by Deas & Wilcox [51], where grouping two vertical lines into an object caused a reduced perception of depth. Such effects could be caused by mechanisms that average across objects, as we suggested here. Pizlo *et al.* [52] found a similar (non-stereo) effect: that the grouping of separated line elements in a Necker cube affected the perceived shape of the object. In both these studies, grouping elements into an object changed the perceived depth, in agreement with our results, suggesting that the visual system is segregating objects before averaging to enhance depth signal strength within an object.

### (d) Stereoscopic camouflage

Finally, we would like to provide some speculations about camouflage. Julesz [1] suggested that stereoscopic vision may have evolved to break camouflage. If this is so, this may have led to an evolutionary arms race where prey animals may themselves have evolved to ‘break’ those stereoscopic camouflage-breaking properties. There is some evidence that prey are camouflaged in a way that disrupts monocular shape-from-shading cue, via an effect called counter-shading [2–4,52,53]. Predator visual systems may use disparity to break that kind of camouflage, and their visual systems may be constrained to first segregate objects and then average. If so, there could be many possibilities for prey animals to also camouflage themselves against stereoscopic observers. For example, an animal could reduce its apparent depth by changing sharp edges in its outline to smooth edges (similar to our smooth stimuli) that merge continuously into the background. This change of edge profile would result in a reduction in perceived depth and could cause the animal to become harder to detect.

Second, a common form of camouflage includes the use of false borders to make the outer edges of an animal ‘break up’ into many separate sections [2,53,54]. If depth averaging occurred after these separate areas were segregated into different objects, then the depth might be averaged over the false, broken up borders. This could lead to several different depth-plateaus being perceived, thus making recognition of a single, continuous prey animal much more difficult. In future studies, we intend to investigate if these forms of potential stereoscopic camouflage do indeed work.

## 5. Summary and conclusion

Participants were unable to correctly estimate displayed peak depth within an object with continuous depth edges. Rather, perceived peak depth was reported as being lower than displayed: thus, the object appeared flatter. HOs were found to have no impact on the perceived depth in our stimuli. The flattening is consistent with averaging over a region that is defined by object segregation, in this case the half-depth of the object. This potentially allows for stereoscopic camouflage, hiding the actual peak depth of an object by deceiving the viewer into perceiving the object as flatter than it truly is.

## Supplementary Material

Supplementary Materials

## Endnote

1 We backed this up by confirming our assumption using a simple cross-correlation disparity extraction model, operating over a number of spatial scales, see the modelling section.

## Data accessibility

Code was written using MathWorks^®^ MATLAB^®^ programming environment, see http://uk.mathworks.com/products/matlab/, with the Psychophysics Toolbox [30,31] and analysed using the Palamedes Toolbox [35,36]. The TNO stereotest is sold by Richmond Products. The datasets supporting this article have been uploaded as part of the electronic supplementary material.
