# Supplementary material for: Depth perception in disparity-defined objects: finding the balance between averaging and segregation
Source: Philos Trans R Soc Lond B Biol Sci. 2016 Jun 19;371(1697):20150258. doi: 10.1098/rstb.2015.0258 (PMC4901452; doi:10.1098/rstb.2015.0258)
Supplement: Supplementary Materials [file rstb20150258supp1.pdf]

Supplementary material for Cammack P and Harris JM. 2016 Depth perception in disparity-defined objects: finding the balance between averaging and segregation. *Phil. Trans. R. Soc. B.* doi: 10.1098/rstb.2015.0258

Section 1: Model Details

Model Details: Square

This model tested the hypothesis that fall in perceived peak disparity for smoother objects is caused by an averaging mechanism within the segregated region of the object. As the object was approximately square, we designed the model to calculate the average disparity within the square region centred on the centre of the stimulus. We called the square region the 'window', and iterated though a large number of available window sizes to find the window size whose average disparity best fitted the perceived peak depth of human observers at all four smoothness coefficients. This model was run individually on each participant.

Note: the equations used here are in full expanded form as used in calculating the integrals, not the (slightly) simplified form as presented in the main manuscript.

Experiment 1

We assumed that as the standard patch was flat with sharply defined edges, and was displayed identically in each trial then it would be perceived as having a constant peak depth. Therefore we did not model the perceived depth of the standard patch.

The shape of the test patch is defined by Eq. 1. For the test patch we predict that the perceived peak disparity of the object will be equal to the average disparity over a square window $x_2 - x_1$ by $y_2 - y_1$ centred on the peak disparity. The shape of the smooth function describing disparity at each point in the object is δ_{shape} and for the first experiment is defined as:

$$\delta_{shape}(x,y) = 0.25d_p\left(\tanh\left(\sigma^{-1}\left(x-\frac{w}{4}\right)\right)-\tanh\left(\sigma^{-1}\left(x-\frac{3w}{4}\right)\right)\right)\left(\tanh\left(\sigma^{-1}\left(y-\frac{h}{4}\right)\right) - \tanh\left(\sigma^{-1}\left(y-\frac{3h}{4}\right)\right)\right) \quad \text{Eq. 1}$$

Where d_p is the peak disparity of the object, σ is the smoothness coefficient and x and y are the coordinates of a given point. w and h are the width (x direction) and height (y direction) of the patch. Here $w = h$.

The simplest way to calculate the total disparity enclosed within the averaging window δ_{window} is to integrate δ_{shape}:

$$\delta_{window} = \int_{x_1}^{x_2} \int_{y_1}^{y_2} \delta_{shape}(x, y)\, dy\, dx$$

$$= 0.25 d_p \int_{x_1}^{x_2} \left(\tanh\left(\sigma^{-1}\left(x - \frac{w}{4}\right)\right) - \tanh\left(\sigma^{-1}\left(x - \frac{3w}{4}\right)\right)\right) dx \times \int_{y_1}^{y_2} \left(\tanh\left(\sigma^{-1}\left(y - \frac{h}{4}\right)\right) - \tanh\left(\sigma^{-1}\left(y - \frac{3h}{4}\right)\right)\right) dy \qquad \text{Eq. 2}$$

The equation is separable into symmetrical x and y components. Taking the first integral we obtain:

$$\int_{x_1}^{x_2} \left(\tanh\left(\sigma^{-1}\left(x - \frac{w}{4}\right)\right) - \tanh\left(\sigma^{-1}\left(x - \frac{3w}{4}\right)\right)\right) dx$$

$$= \sigma \ln\left(\frac{\cosh\left(\sigma^{-1}\left(\frac{w}{4} - x_2\right)\right) \operatorname{sech}\left(\sigma^{-1}\left(\frac{w}{4} - x_1\right)\right)}{\cosh\left(\sigma^{-1}\left(\frac{3w}{4} - x_2\right)\right) \operatorname{sech}\left(\sigma^{-1}\left(\frac{3w}{4} - x_1\right)\right)} \right) \qquad \text{Eq. 3}$$

We now define a function $f(x_2, x_1)$ such that:

$$f(x_2, x_1) = \ln\left(\frac{\cosh\left(\sigma^{-1}\left(\frac{w}{4} - x_2\right)\right) \operatorname{sech}\left(\sigma^{-1}\left(\frac{w}{4} - x_1\right)\right)}{\cosh\left(\sigma^{-1}\left(\frac{3w}{4} - x_2\right)\right) \operatorname{sech}\left(\sigma^{-1}\left(\frac{3w}{4} - x_1\right)\right)} \right) \qquad \text{Eq. 4}$$

Substituting this back into equation 2 for δ_{window} we obtain:

$$\delta_{window} = 0.25 \delta_p \sigma^2 f(x_2, x_1) f(y_2, y_1) \qquad \text{Eq. 5}$$

To translate this into the prediction of the peak depth all we need is to divide by the area of the window:

$$\delta_{prediction} = \frac{\delta_{window}}{4(x_2 - x_1)(y_2 - y_1)}$$

$$\frac{\delta_p \sigma^2 f(x_2, x_1) f(y_2, y_1)}{4(x_2 - x_1)(y_2 - y_1)} \qquad \text{Eq. 6}$$

This gives us the predicted peak depth for an arbitrary window size $(x_2 - x_1)$ by $(y_2 - y_1)$ where $y_1 = x_1$ and $y_2 = x_2$ (we only consider square windows) and smoothness coefficient σ. We calculated the predicted peak depth for a range of window sizes and all smoothness coefficients used in Experiment 1.

For each tested window size, we compared the predicted peak depth at each smoothness coefficient to the results, for each participant. The reduced chi-squared test score was used across all smoothness coefficients to calculate a goodness of fit for each window size. We selected the window-

size that had the minimum reduced chi-squared test score as the best prediction for the individual participant. The reduced chi-squared test was used as it takes the uncertainty in the participant's data at each smoothness coefficient into account, giving less weight to data with greater uncertainty, and therefore enabling a better fit to be produced.

This process was repeated for all participants, until each participant has a predicted window size calculated from the best fit of the model. We analysed the variance of the model from the participant's data using the R^2 test score to give us an idea of how well the model fitted each participant's performance.

Experiment 2

This process can also be repeated for the stimulus in Experiment 2, resulting in:

$$x_2 - x_1 \leq w, \qquad \delta_{prediction} = \frac{f(x_2, x_1)}{2(y_2 - y_1)} \qquad \text{Eq. 7}$$

$$x_2 - x_1 > w, \qquad \delta_{prediction} = \frac{f(x_2, x_1)w}{2(y_2 - y_1)(x_2 - x_1)} \qquad \text{Eq. 8}$$

Due to the symmetrical disparity distribution in both condition 1 and 2, and as the stimulus and window was square this equation holds for both condition 1 and condition 2 (although x and y are switched algebraically).

Fitting was done as with Experiment 1, where a range of window sizes were tested.The window size that best fit all smoothness coefficients simultaneously, according to the reduced chi-squared test score, was considered to be the best fit.

Experiment 3

From Experiment 1, the model delivered an averaging window size within 15% of the stimulus plateau size, for all participants. However, this was also the size of the disparate standard object patch. Experiment 3 was designed to test whether the visual system 'chose' its averaging window on the basis of the stimulus-specific plateau, or using the standard patch size as a template. Thus, we implemented two models, the half-depth averaging model (based on the stimulus plateau size), and the template model (based on the standard stimulus patch size). Note therefore that there are no free parameters in these models. The experiment used a range of plateau sizes so that we could decide which model best fit the human data.

We repeated the logic used above to define the models here:

$$\delta(x,y) = 0.25\,\delta_p \left(\tanh(\sigma^{-1}(x-b)) - \tanh\left(\sigma^{-1}(x-(w-b))\right)\right)\left(\tanh(\sigma^{-1}(y-b)) - \tanh\left(\sigma^{-1}(y-(w-b))\right)\right) \qquad \text{Eq. 9}$$

Where b is the size of the border around the plateau size p:

$$p = w - 2b \quad \text{Eq. 10}$$

Following the same methodology, and defining a function $g(x)$:

$$g(x_2, x_1) = \ln\left(\frac{cosh(\sigma^{-1}(b - x_2))\,sech(\sigma^{-1}(b - x_1))}{\cosh(\sigma^{-1}(w - b - x_2))\,\operatorname{sech}(\sigma^{-1}(w - b - x_1))}\right) \quad \text{Eq. 11}$$

$$\delta_{prediction} = \frac{\delta_p \sigma^2 g(x_2, x_1) g(y_2, y_1)}{4(x_2 - x_1)(y_2 - y_1)} \quad \text{Eq. 12}$$

For the **half-depth model**, where averaging is dependent on the size of the plateau of the smooth stimulus, we used the size of the plateau for each stimulus in the experiment as the window size. Performance was analysed using the R^2 test statistic in comparison to the participants data.

$$x_2 = \frac{w + p}{2}$$

$$x_1 = \frac{w - p}{2}$$

For the **template model** averaging was dependent on the size of the standard stimulus patch, we therefore chose a constant window size for all shapes of the smooth stimulus. Again, this left us with no free parameters to fit, so we simply tested performance using the R^2 test statistic. Here, we used:

$$x_2 = \frac{3w}{4}$$

$$x_1 = \frac{w}{4}$$

Model Details: Circular

This model tested whether the shape of the averaging window was important for the model used in Experiment 1. In order to fit a circular window to the stimulus in Experiment 1, two different approaches were used. Due to the complexity of the integral from of the function in polar coordinates, we report a model based on using a matrix of disparity values D from Eq. 1. The (i, j)th position of each element of the matrix corresponded to an (x, y) position on the stimulus, the magnitude of the element corresponded to the disparity at that point. We then summed all the values within a circular window of radius r with an origin in the centre of the stimulus, to obtain the total disparity enclosed by the circular region. Dividing by the total number of values within this circle delivered the predicted disparity δ_{circle}. MATLAB® results for each fitted radius were taken and compared to the integral form run in Wolfram Mathematica®, and found to be within 0.1arcmin.

This model was implemented by generating a second matrix A such that $A_{ij} = 1$ if the element was within the circular window and $A_{ij} = 0$ if it was outside:

$$A_{ij} = \begin{cases} 1 \; if \; \sqrt{\left(x - \frac{w}{2}\right)^2 + \left(y - \frac{h}{2}\right)^2} \leq r^2 \\ 0 \; if \; \sqrt{\left(x - \frac{w}{2}\right)^2 + \left(y - \frac{h}{2}\right)^2} > r^2 \end{cases} \qquad \text{Eq. 13}$$

The Hadamard product of the disparity matrix D and the matrix A was then taken. When divided by the sum of A (this equals the number of non-zero elements in $D \circ A$), we obtained the average disparity in within the circle.

$$\delta_{circle} = \frac{\sum_{i,j}(D \circ A)}{\sum_{i,j}(A)} \qquad \text{Eq. 14}$$

An analogous model using this method and a square window was also developed, and performed fits to within one pixel of window size (1.07min arc) of the original integral model, although with a significantly inferior runtime and lower accuracy than the integral method presented above.

Section 2: Model results

Experiment 1

See the main body of the paper for the fitting parameters for the square window model in Experiment 1; Figure 3. See Supplementary Supplementary Figure **1**, below, for best-fit conditions for the circular model applied to each participant's data. The figure shows that the model is clearly a poor fit to participant performance. Analysis of R^2 (see Table 1) confirmed this: an R^2 of -1 implies that the function is better fitted with a straight line. We therefore rejected this circular window model in favour of the square-based model presented in the main text.

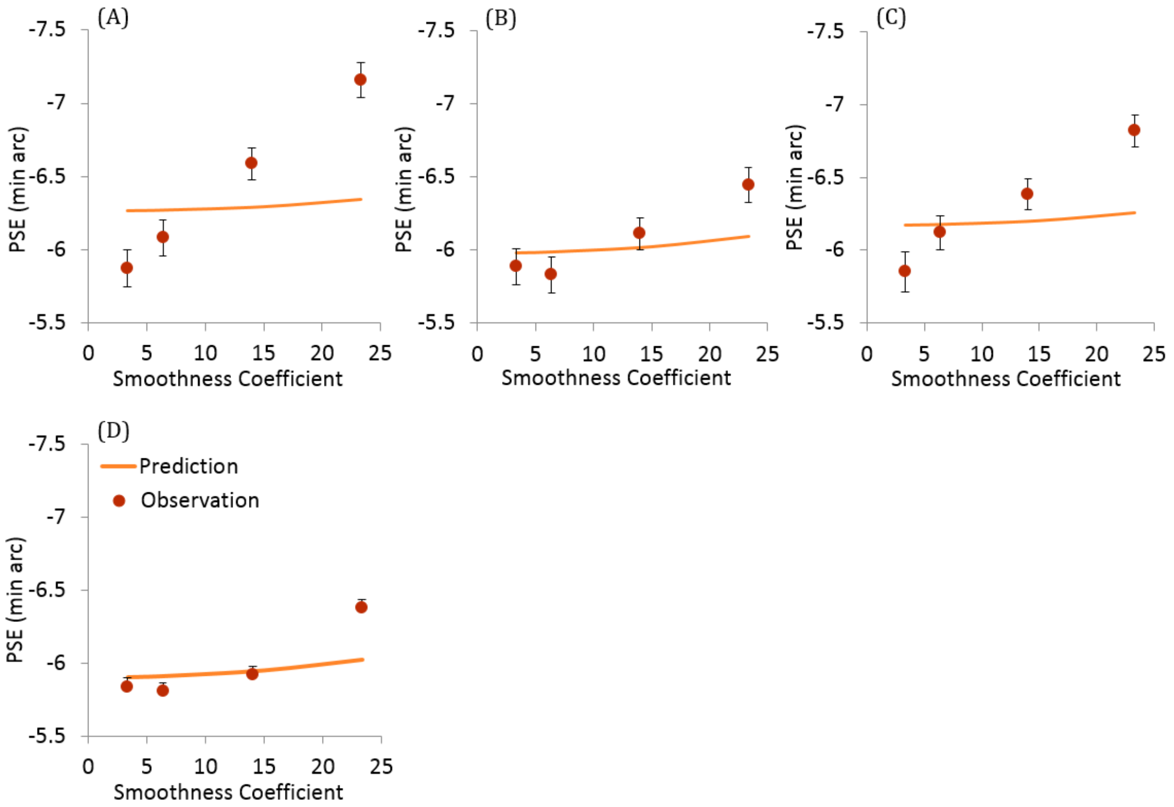

Supplementary Figure 1: Best fit conditions for the participants (a), (b) (c) and (d) for the circular model on data from Experiment 1 Each graph shows data and model fit from a single participant. Error bars are one standard error.

Participant	Window Size (min arc)	R^2
(a)	165.3	-239
(b)	146.0	-20
(c)	158.3	-89
(d)	139.5	-15
Average	152.4	-91

Supplementary Table 1: fitting data for a circular window for model for experiment 1. Note that a value of $\mathrm{R}^2 < -1$ indicates that the data is better fitted by a straight line than the proposed function.

Experiment 2

The window model was also applied to Experiment 2 as shown in Supplementary Figure 2, using the integral of the function that generated the Experiment 2 stimulus (calculated above). The average window size was 163.2 min arc, with a standard deviation of 10min arc (see Supplementary Table 2).

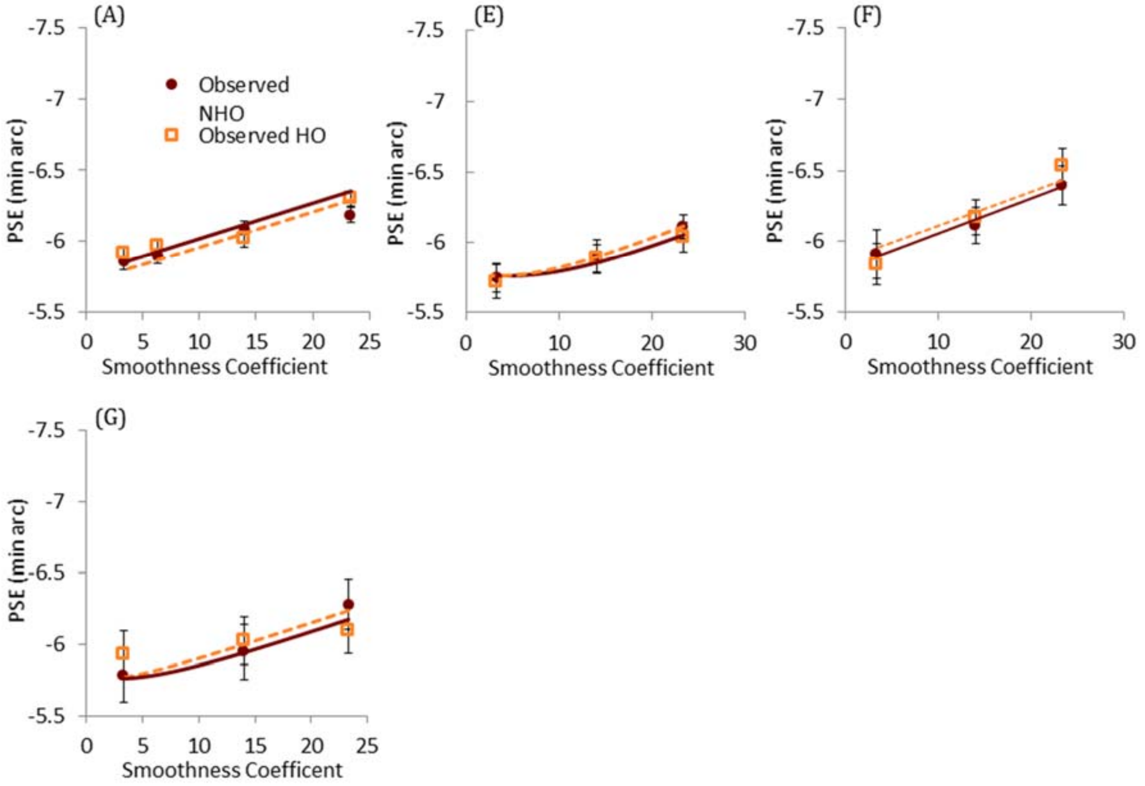

Supplementary Figure 2: Best fits of the model for the square window model on data from Experiment 2. NHO was the stimulus with no half occlusions, HO was the stimulus with half occlusions. Error bars are one standard error.

Participant	Condition	Window Size (min arc)	Window Size (% of sharp-edged patch)	R^2
(a)	NHO	151.7	89	1
(a)	HO	145.1	85	0.94
(b)	NHO	172.2	101	0.98
(b)	HO	173.3	101	0.79
(c)	NHO	163.2	95	0.96
(c)	HO	157.2	92	0.53
(d)	NHO	167.8	98	0.98
(d)	HO	171.4	100	0.88
Average	NHO	164.2	96	98
Average	HO	162.3	94	87*
Average	Total	163.2	95	93*

Supplementary Table 2: Model fitting parameters for the four participants in Experiment 2 and both conditions. Condition NHO had no half occlusions, condition HO had half occlusions. *Average $\boldsymbol{R^2}$ excludes participant (C) condition 2.

Note that the model fit for the HO stimulus for participant (C) had a very low r-squared at 0.53 (see Supplementary Table 2 for full data). Inspection of Supplementary Figure 2 (C) revealed that the second condition (HO, red squares) showed a remarkably large error bar compared to the other participants, implying that this participant struggled on this particular condition. In Supplementary Figure 2 (c) it is clear that the fit was significantly different to the experimental data for this participant. However, all other fits to experiment 2 average out with $R^2 = 0.93$ and a standard deviation of 0.07, corroborating the results from running the model on Experiment 1.

Section 3: Psychometric Functions

Here we include the psychometric function plots for all participants used in the paper. We also include the fits for participants whose data was recorded, but were excluded from further study, which we label as ~1 to ~4.

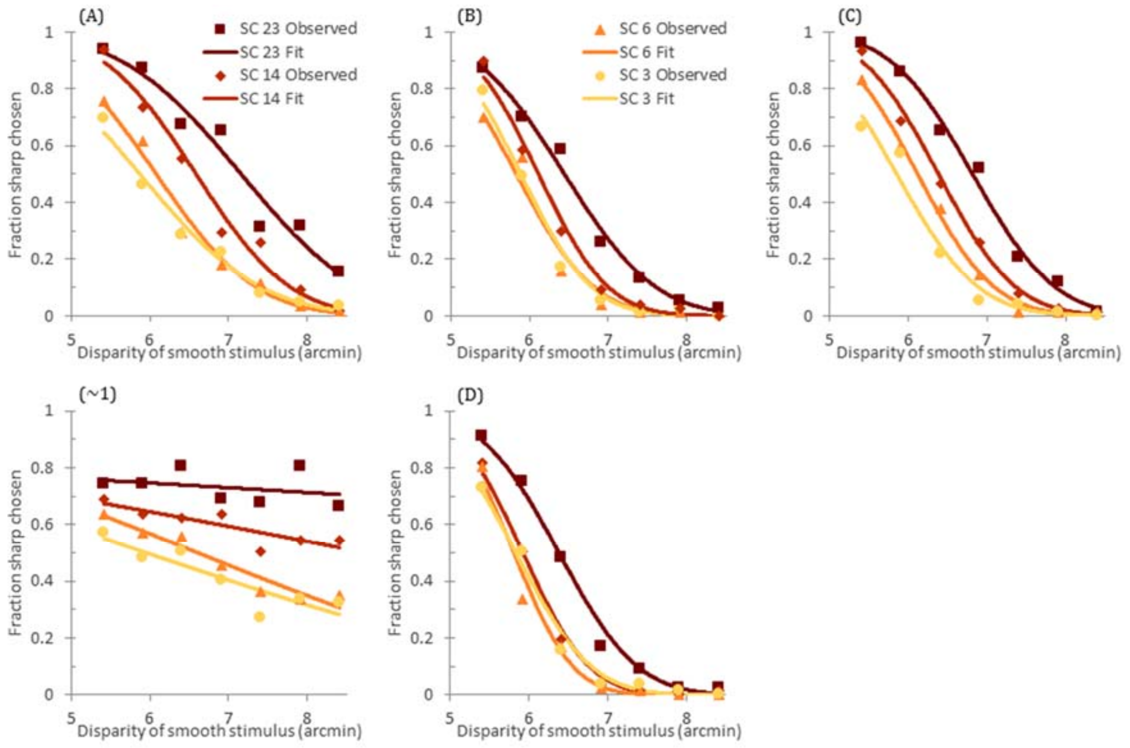

Supplementary Figure 3: Psychometric functions for all participants for Experiment 1.

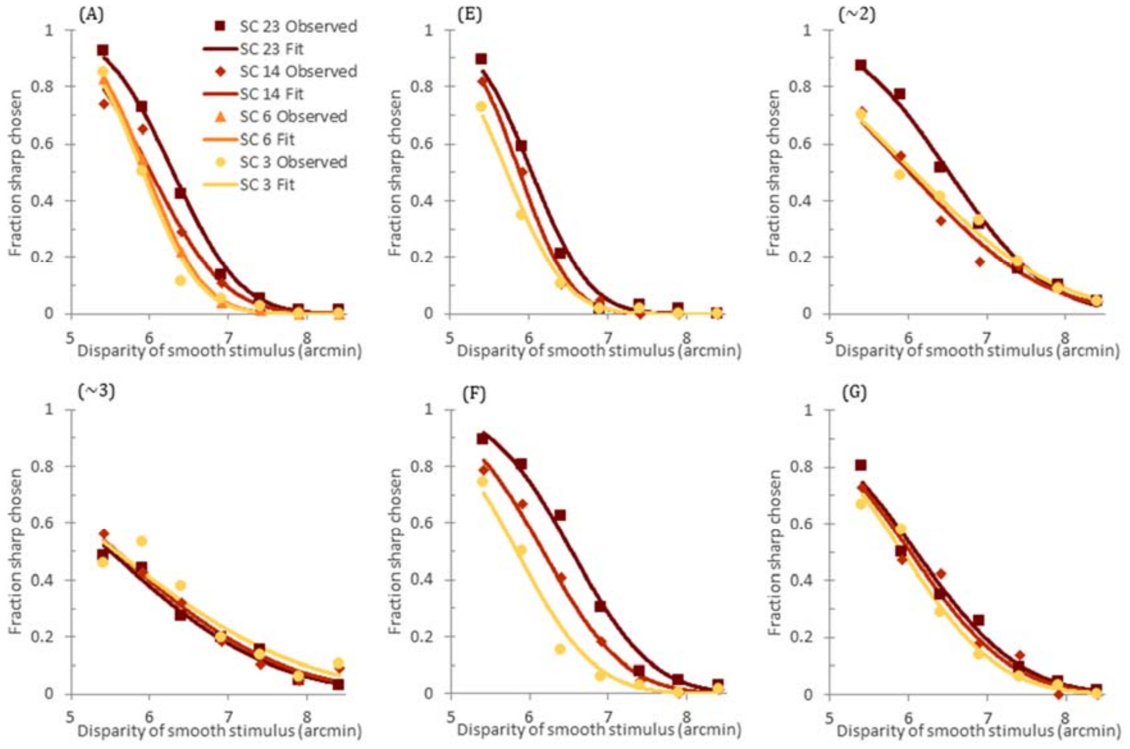

Supplementary Figure 4: Psychometric functions for all participants for Experiment 2, half occlusion (HO) condition.

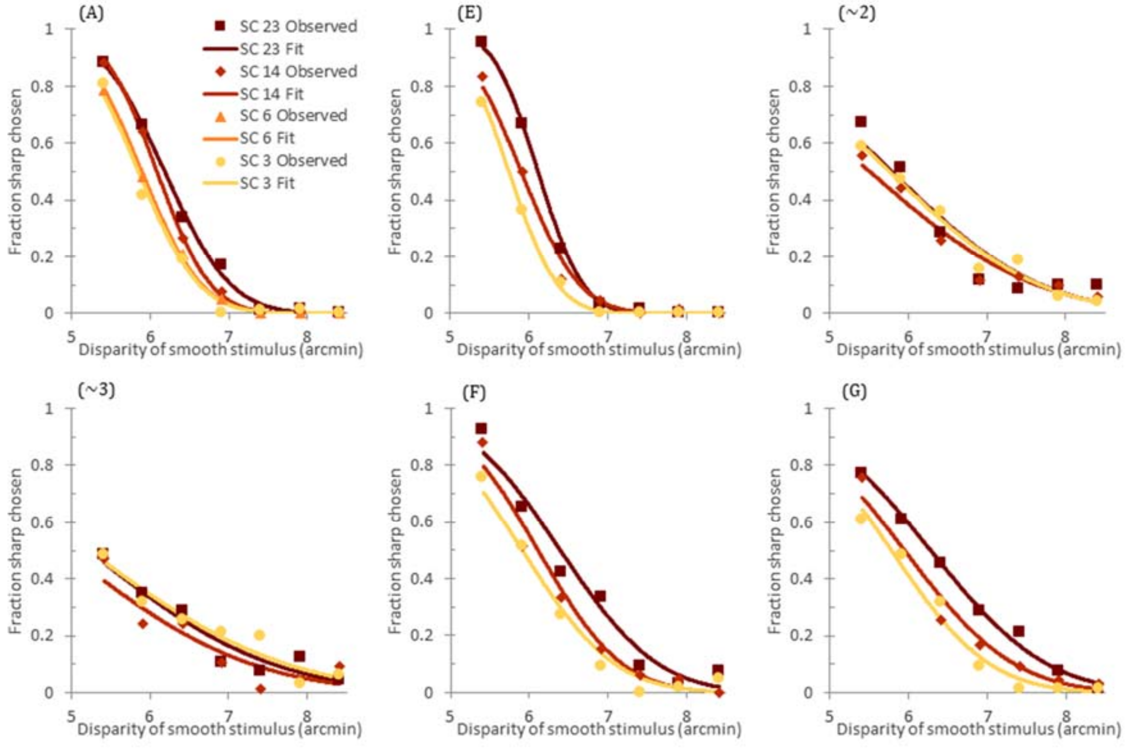

Supplementary Figure 5: Psychometric functions for all participants for Experiment 2, no half occlusion (NHO) condition.

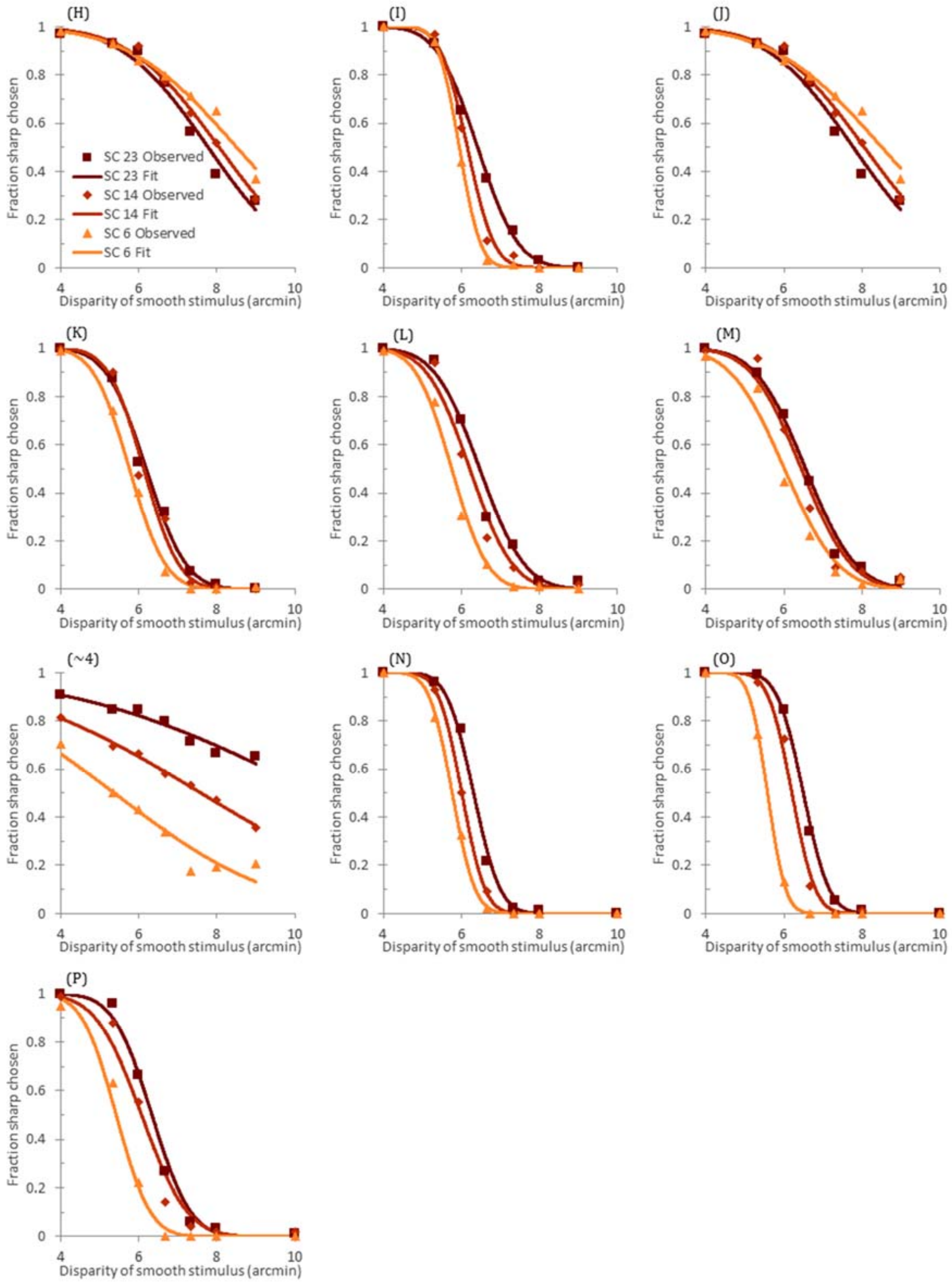

Supplementary Figure 6: Psychometric functions for all participants for Experiment 3.

© The Authors under the terms of the Creative Commons Attribution License http://creativecommons.org/licenses/by/3.0/, which permits unrestricted use, provided the original author and source are credited.
